# Factors Associated with Serum 25-Hydroxyvitamin D Concentration in Two Cohorts of Pregnant Women in Southern Ontario, Canada

**DOI:** 10.3390/nu11010123

**Published:** 2019-01-09

**Authors:** Maude Perreault, Caroline J. Moore, Gerhard Fusch, Koon K. Teo, Stephanie A. Atkinson

**Affiliations:** 1 Department of Pediatrics, McMaster University, Hamilton, L8S 4L8, Canada; perream@mcmaster.ca (M.P.); camoore@mcmaster.ca (C.J.M.); gefusch@mcmaster.ca (G.F.); 2 Department of Medicine (Cardiology), McMaster University, Hamilton, L8S 4L8, Canada; teok@mcmaster.ca

**Keywords:** serum 25-OHD, pregnancy, developmental origins of health and disease, bone health

## Abstract

Vitamin D deficiency in pregnancy is widely reported, but whether this applies in North America is unclear since no population-based surveys of vitamin D status in pregnancy exist in Canada or the United States. The objectives were to assess (i) the intake and sources of vitamin D, (ii) vitamin D status, and (iii) factors associated with serum 25-hydroxyvitamin D (25-OHD) concentration in two cohorts of pregnant women from Southern Ontario, Canada, studied over a span of 14 years. Maternal characteristics, physical measurements, fasting blood samples and nutrient intake were obtained at enrolment in 332 pregnant women from the Family Atherosclerosis Monitoring In early Life (FAMILY) study and 191 from the Be Healthy in Pregnancy (BHIP) study. Serum 25-OHD was measured by LC/MS-MS. The median (Q1, Q3) total vitamin D intake was 383 IU/day (327, 551) in the FAMILY study and 554 IU/day (437, 796) in the BHIP study. Supplemental vitamin D represented 64% of total intake in participants in FAMILY and 78% in BHIP. The mean (SD) serum 25-OHD was 76.5 (32.9) nmol/L in FAMILY and 79.7 (22.3) nmol/L in BHIP. Being of European descent and blood sampling in the summer season were significantly associated with a higher maternal serum 25-OHD concentration. In summary, health care practitioners should be aware that vitamin D status is sufficient in the majority of pregnant Canadian women of European ancestry, likely due to sun exposure.

## 1. Introduction

Adequate maternal vitamin D status is critical to pregnancy health outcomes and the vitamin D status of the infant at birth, and may program for bone development in childhood and later life [1,2,3]. Vitamin D is essential for bone mineralization, proper bone accretion and growth of the fetus during pregnancy [4]. Systematic reviews of randomized studies identified the effects of maternal vitamin D supplementation on reducing low birth weight prevalence and improving infant growth, with some indications of its potential benefit on pregnancy complications such as pre-eclampsia [5,6,7]. Maternal vitamin D status in pregnancy has also been positively associated with bone health outcomes in infants [8], children [9,10] and adolescents [11].

Given the potential health benefits of vitamin D, supplementation has gained popularity in the general public over the last decade. The dose in common brands of prenatal multivitamins in Canada has increased from 300 to 600 IU/pill on average over the years, but doses of over-the-counter multi-nutrient supplements range from 200 to 1000 IU/tablet, and single vitamin D supplements are available in doses of 1000–10,000 IU/tablet. As well, the consumption of vitamin D fortified products is gaining popularity in the market as pregnant women become aware of the possible benefits of vitamin D during and after pregnancy.

Despite the potential importance of maternal vitamin D status on the health outcomes of mother and child, no population-based data exist as pregnant women have not been sampled in the nutrition and health surveys in Canada or the United States to date. Claims of a “pandemic” or a high prevalence of vitamin D deficiency in pregnant and lactating women in Canada [12] are not founded on population-based surveys, but rather, cite literature primarily from Afro-American and Indigenous groups living in Canada. In a single US study [13], it was postulated that intakes of 4000 IU of vitamin D per day during pregnancy are required to optimize the production of 1,25-dihydroxyvitamin D (1,25-OH_2_D) and cord blood 25-hydroxyvitamin D (25-OHD). However, no study to date has demonstrated that vitamin D intakes in pregnancy of >400 up to 4000 IU/day result in any functional benefits to mother or infant. Thus, in the recently revised Dietary Reference Intakes (DRI) [14], the Estimated Average Requirement (EAR) for vitamin D in pregnancy is the same as for non-pregnant women at 400 IU per day. This recommendation was confirmed by the Scientific Advisory Committee on Nutrition in the United Kingdom [15], as well as by the European Food Safety Authority in Europe [16].

The present study was undertaken with the objective to assess (i) the intake and sources of vitamin D, (ii) the vitamin D status, and (iii) the factors associated with maternal serum 25-OHD concentration as a measure of vitamin D status in two cohorts of pregnant women living in Southern Ontario, Canada, studied over a span of 14 years. 

## 2. Materials and Methods

### 2.1. Study Design

Pregnant women enrolled in the FAMILY and BHIP studies were included, both of which were conducted in accordance with the Declaration of Helsinki. The Family Atherosclerosis Monitoring In early Life (FAMILY) study was a longitudinal, prospective birth cohort study designed to investigate the determinants of obesity, type 2 diabetes and cardiometabolic traits early in life [17]. A total of 857 pregnant women were recruited through three hospitals in Hamilton and Burlington, Ontario, Canada between the years of 2002–2009. For this ancillary study on factors associated with maternal serum 25-OHD concentration, separate ethics approval was granted for the assessment of vitamin D status and bone health in subjects of the FAMILY study by the Research Ethics Board at Hamilton Health Sciences/McMaster University (REB #02-060). Participants gave informed written consent for this sub-study. The Be Healthy in Pregnancy (BHIP) Study is an ongoing randomized controlled trial (RCT; Clinical Trials Ref: NCT01693510) [18] for which the primary research objective is to determine whether introducing a structured and monitored nutrition and exercise program in early pregnancy, compared to standard prenatal care, will increase the number of women attaining gestational weight gain within the Institute of Medicine (IOM), Health and Medicine Division recommendations for their pre-pregnancy body mass index (BMI) category [19]. The present analysis included data obtained at baseline prior to randomization. Between the years 2012–2018, 274 healthy pregnant women were recruited from health care clinics in Hamilton, Burlington and London, Ontario. Informed written consent was obtained upon study enrolment. Ethics approval was obtained from the Research Ethics Boards of Hamilton Health Sciences (REB Project#12-469), Western Ontario in London (HSREB 103272), and Joseph Brant Hospital in Burlington (JBH 000-018-14), all in Southern Ontario, Canada.

### 2.2. Maternal Data Collection

Maternal demographics, pregnancy history, fasting blood samples and physical measurements were obtained from each participant upon study entry. For the FAMILY study, the information and blood samples were obtained between 24 and 36 weeks of gestation, while for the BHIP study, they were collected between 12 and 17 weeks of gestation. Maternal height and weight were measured, gestational weight gain was self-reported and pre-pregnancy BMI was calculated. Ethnicity, education level and annual household income were self-reported. Maternal health behaviours were self-reported using questionnaires. For the FAMILY study, participants completed a validated semi-quantitative multi-ethnic food frequency questionnaire (FFQ) including supplements [20,21] Nutrient composition was calculated as previously described [22], excluding records where the FFQ was <50% incomplete, or with implausible dietary intakes (<500 or >4500 kcal/day). For the BHIP study, participants completed diet records for three consecutive days (two weekdays and one weekend day) including both foods and supplements, as used previously in pregnant women [23]. Participants were asked to weigh the foods eaten when possible, using household measures such as cups/spoons when weight was not able to be determined. No diet records with implausible intakes were found in the BHIP study. Diet records were analyzed using Nutritionist Pro diet analysis software (Version 5.2, Axxya Systems, Stafford, TX, USA), and the Canadian Nutrient File (version 2015) to obtain daily intake of vitamin D. For the FAMILY study, dairy products were classified as low fat (≤2% fat) or regular fat (≥3.25% fat) dairy products. For the BHIP study, the categories were milk (low and regular fat combined), yogurt (low and regular fat combined), and other dairy products (i.e., regular fat sour cream, cream, cheese, and ice cream). In Canada, all milk and margarine products are fortified with vitamin D_3_ by law. Yogurt and other dairy products are sometimes made from vitamin D_3_ fortified milk, and this is noted on the label. Exercise was self-reported by participants in both studies. In the FAMILY study, participants completed a validated questionnaire [24], and were categorized as currently exercising or not. In the BHIP study, participants reported exercising or not at recruitment by completing the Physical Activity Readiness Medical Examination (PARmed-X) for Pregnancy [25]. 

### 2.3. Vitamin D Analysis

Serum 25-OHD (D_2_ and D_3_ isomers) was measured by ultra-performance liquid chromatography tandem mass spectrometry (UPLC-MS/MS) using the Waters application note 720002748 [26] with a modified sample preparation that included a saponification step [27]. Saponification prevents fat droplet formation in the supernatant after extraction, which can occur in plasma with a high triglyceride concentration. Saponification converts triacylglycerides into water-soluble fatty acid soaps. This is particularly important as circulating lipids can be elevated in pregnant women [28]. In short, 150 µL of serum and 10 µL of internal standard solution (800 nmol/L, 25-OHD_3_-d_6_; 99% pure; Medical Isotopes, Pelham, NH, USA) were vortexed and 50 µL of methanol (Fisher Scientific, Ottawa, Ontario, Canada), 100 µL of ascorbic acid (20% *w*/*v*, (>99.9% pure); Sigma Aldrich, Oakville, Canada) and 40 µL of potassium hydroxide (45% *w*/*v*; Fluka Analytical, Ronkonkoma, NY, USA) were added. Samples were placed in a 75 °C hot water bath for 20 minutes. After cooling down to room temperature, the mixture was extracted with 500 µL of heptane (Fluka Analytical, Ronkonkoma, NY, USA). The organic phase was evaporated under a gentle stream of nitrogen, and reconstituted in 75 µL of methanol (MS grade, Fisher Scientific, Ottawa, Ontario, Canada). The accuracy and precision of the LC-MS/MS method for measuring 25-OHD_2_ and 25-OHD_3_ was evaluated using National Institute of Standards and Technology (NIST) Standard Reference Material 972a (Bureau of Standards, Washington, DC, USA). 25-OHD_3_/25-OHD_2_ serum controls purchased from BioRad (Munich, Germany) served as a daily quality control. A Waters Acquity UPLC/TQD system was used with an Acquity UPLC BEH C18 column. The transitions m/z 401/383 for 25-OHD_3_, 407/389 for 25-OHD_3_-d_6_, and 413.5/395.3 for 25-OHD_2_ were used for quantification.

### 2.4. Statistical Analysis

Statistical analysis was performed using JMP® 9.0 (Version 9.0.1, SAS Institute Inc., Cary, NC, USA) and GraphPad Prism (Version 7, La Jolla, CA, USA). Descriptive statistics were computed by calculating the means and standard deviations of normally distributed continuous data; medians and quartiles (Q1 and Q3) for non-normally distributed continuous data; and counts and percentages for categorical data. *T*-tests were performed to compare groups and significance was established at *p* < 0.05. Analysis of variance (ANOVA) was performed to compare 25-OHD concentrations in women of different pre-pregnancy BMI categories. Significance was established at *p* < 0.05. Mean values are given as means ± standard deviations if not stated otherwise. To determine which factors were associated with maternal serum 25-OHD concentration, we conducted a multivariable linear regression analysis. The variables of interest included in our multivariable regression were decided a priori based on clinical rationale and evidence from the literature. The non-standardized regression coefficients and their corresponding 95% confidence intervals (CIs) and *p*-values for the multivariable analyses are presented.

## 3. Results

### 3.1. Demographics and Physical Characteristics

A total of 332 participants from the FAMILY study and 191 from the BHIP study were included in this report as they had available maternal serum samples analysed for 25-OHD. Participants of the FAMILY study were enrolled at a median of 28 weeks gestation, while the BHIP study participants were enrolled at a median of 13 weeks gestation. Half of the FAMILY participants (54%) were enrolled by 2006 and all by 2009, while most participants (96%) of the BHIP study were enrolled between 2013 and 2017. The mean (SD) age of the participants was significantly higher in the FAMILY study than in the BHIP study (32.5 (4.7) vs. 31.2 (3.9) years; *p* = 0.001). Pre-pregnancy BMI was not statistically different between studies (Table 1). According to the pre-pregnancy BMI data, about half of the participants had normal weight, while half were categorized as overweight or obese upon entering pregnancy (Table 1). Most participants were of European ancestry, had a tertiary level of education, and were currently exercising. Few participants in the FAMILY study and none in the BHIP smoked during pregnancy, the latter because it was an exclusion criterion.

### 3.2. Intake of Vitamin D in Both Studies: Trend Over 10 Years

**Figure 1:**
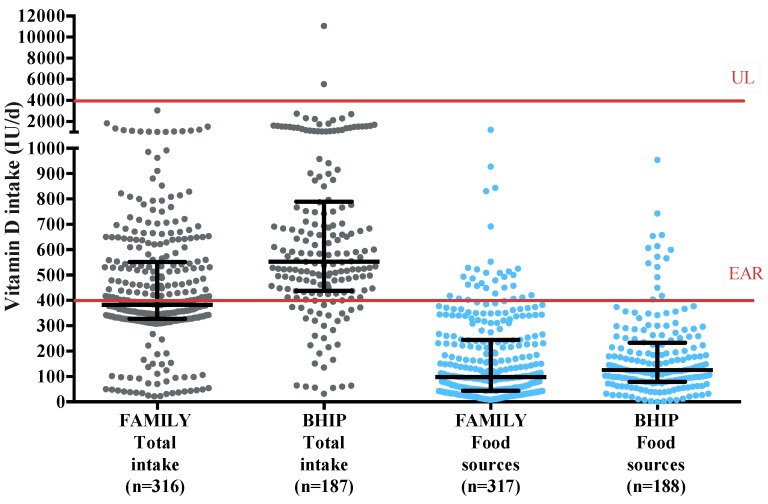
Maternal dietary intake of vitamin D of participants in the FAMILY and BHIP studies. Median and interquartile ranges are displayed for total intake and food sources contribution to total vitamin D. — Vitamin D recommendations for pregnancy: Estimated Average Requirement (EAR) = 400 IU/day; Tolerable Upper Intake Level (UL) = 4000 IU/day [14].

The median (Q1, Q3) total vitamin D intake in the FAMILY study was 383 IU/day (327, 551) with the highest intake being 3050 IU/day (Figure 1). The median total vitamin D intake in the BHIP Study was 554 IU/day (437, 796) with the highest intake being 11,062 IU/day. Intakes of vitamin D met the EAR of 400 IU/day in 43% of participants in the FAMILY study and 80% in the BHIP study. Vitamin intake from food sources alone met the EAR in only 9% of participants in both the FAMILY and BHIP studies (Figure 1). No participants in the FAMILY study exceeded the Tolerable Upper Intake Level (UL) of 4000 IU/day, and only three participants in the BHIP (2%) exceeded the UL. Supplement intake represented 64% (289 IU/day) of total intake in the FAMILY study and 78% (629 IU/day) in the BHIP study. Supplements containing vitamin D, mostly prenatal multivitamins, were consumed by 87% of participants in the FAMILY study. The median (Q1, Q3) intake of vitamin D from multivitamins was 300 IU/day (300, 300) but ranged from 0 to 3000 IU/day. In the BHIP study, 92% of participants were consuming supplements containing vitamin D. The median (Q1, Q3) intake of vitamin D from multivitamins was 400 IU/day (400, 600), ranging from 0 to 11,000 IU/day for some participants. 

### 3.3. Maternal Serum 25-OHD Concentration during Pregnancy

**Figure 2:**
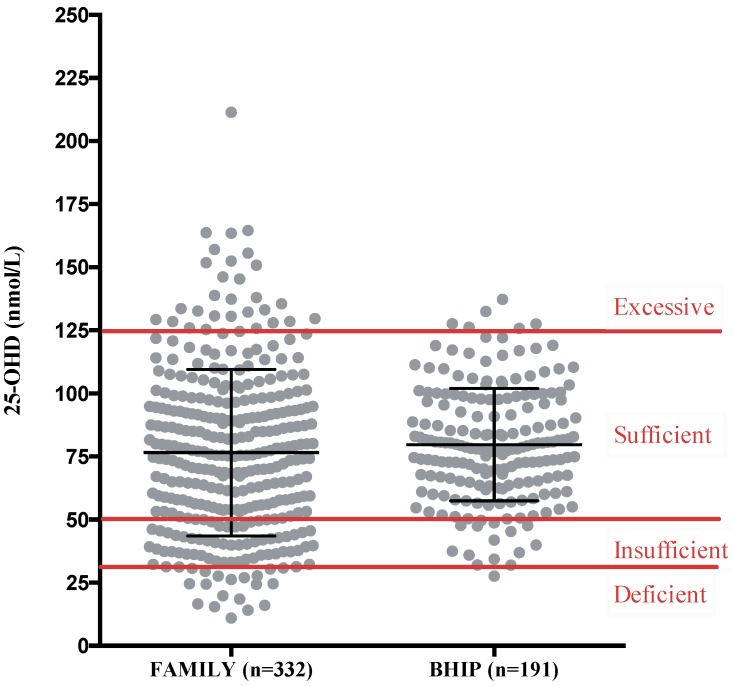
Maternal serum 25-OHD concentration (mean, SD) in participants from the FAMILY and BHIP studies in comparison to the recommendations by the Institute of Medicine [14]; — <30 nmol/L deficient, 30–50 nmol/L insufficient, ≥50 nmol/L sufficient, >125 nmol/L excessive.

**Figure 3:**
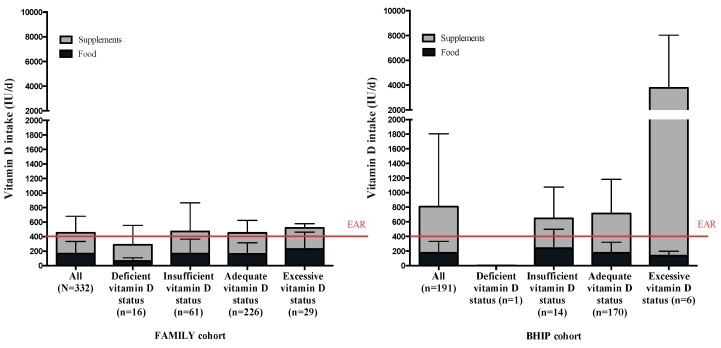
Total vitamin D intake in the FAMILY and BHIP studies, by maternal serum 25-OHD concentration. Mean and standard deviation are displayed. The Estimated Average Requirement (EAR) is indicated by — [14].

The mean serum 25-OHD concentration was in the optimal range (serum 25-OHD 50–125 nmol/L [14]). The isomer 25-OHD_2_ was detected in only 6% of participants in the BHIP study (data not available for the FAMILY study) at a concentration of 0.51 ± 2.89 nmol/L (mean ± SD); thus, the D2 isomer did not contribute significantly to the overall total serum 25-OHD. Accordingly, the total circulating 25-OHD is a reflection of 25-OHD_3_. Serum 25-OHD concentrations did not differ between women with different pre-pregnancy BMI values in either study (Table 2). The season that blood was drawn was significantly associated with 25-OHD concentrations in both studies, where blood samples collected in summer had higher serum 25-OHD than in winter (*p* = 0.001 in FAMILY and *p* = 0.002 in BHIP). The threshold representing a sufficient 25-OHD concentration of 50 nmol/L as set by the IOM [14] was met or exceeded by 77% of participants in the FAMILY study and 93% of participants in the BHIP study (Figure 2). The range of maternal 25-OHD concentrations was greater in the FAMILY study compared to in the BHIP study; 5% of participants in the FAMILY study had serum 25-OHD in the deficient range (<30 nmol/L) and 9% of participants exceeded 125 nmol/L, the level for excessive circulating 25-OHD. In the BHIP study, only 0.5% of participants were clinically deficient, while 3% surpassed the excessive threshold. No correlation was observed in the FAMILY study between maternal total vitamin D intake and maternal serum 25-OHD concentration (*R*^2^ = 0.01, *p* = 0.09). In the BHIP study, a higher total intake of vitamin D was associated with higher serum 25-OHD (*R*^2^ = 0.11, *p* < 0.0001). For BHIP, supplements contributed an important amount to the total vitamin D intake and contributed to a higher 25-OHD concentration (Figure 3).

### 3.4. Factors Associated with Maternal Serum 25-OHD Concentrations in Pregnancy

For participants enrolled in the FAMILY study, a multivariable analysis demonstrated that a higher maternal 25-OHD concentration was significantly associated with being of European descent, having blood drawn in summer, and having a low pre-pregnancy BMI (Table 3). 

For participants enrolled in the BHIP study, a multivariable analysis revealed that higher maternal 25-OHD concentrations were significantly associated with having blood drawn in summer and vitamin D intake from regular fat dairy products (i.e., sour cream, cream, ice cream, cheese) (Table 3). 

## 4. Discussion

The majority of healthy pregnant women in Southern Ontario in the last 10 years have had sufficient circulating 25-OHD both in early and late pregnancy using the reference cut-off values from the DRI report [14]. While risk for vitamin D deficiency may exist globally [29], this does not appear to apply to pregnant women living in Southern Ontario. Health care practitioners should be aware that in our community, <5% of pregnant women had serum 25-OHD <30 nmol/L, which is similar to what was found in the general Canadian population (4%, defined as <27.5 nmol/L) by the Canadian Health Measures Survey (CHMS) [30]. The overall circulating 25-OHD in the participants in the two combined pregnant cohorts was 77.7 nmol/L, a value slightly higher than the average serum 25-OHD of 69.5 nmol/L reported for females of child-bearing age (20–39 years old) in the CHMS [30], and which is significantly higher than observed in males in the same age category. The latter is likely attributable to higher vitamin supplement intake among females [30]. High intakes of supplements containing vitamin D were observed in our two cohorts of pregnant women where approximately 90% were taking prenatal supplements. Vitamin D supplementation is known to be associated with higher 25-OHD status, but the response can be highly heterogeneous among pregnant women [7,31], including those in our study. Despite the majority of participants taking prenatal supplements in both the FAMILY and BHIP studies, heterogeneity was indicated, in which prenatal supplement intake was not associated with 25-OHD status in FAMILY (*R*^2^ = 0.00, *p* = 0.58) but a modest albeit significant association with 25-OHD status was observed in BHIP (*R*^2^ = 0.11, *p* < 0.0001).

Although the average maternal intake of vitamin D from food did not reach the EAR of 400 IU/day and this was only weakly associated with 25-OHD concentration, the estimated total vitamin D intake from both food and supplements exceeded 400 IU/day in only 45% of participants in the FAMILY study but in 80% of the BHIP participants. Our results are in agreement with data reported from Canadian studies showing that the primary source of oral vitamin D (approximately 60% total intake) is supplements [32,33]. In the Canadian food chain, there are limited natural or fortified food sources of vitamin D. According to our data and those of other Canadian studies [32,33,34,35,36,37], intake of vitamin D supplements is common in pregnancy, and combined with sun exposure, the prevalence of inadequacy of vitamin D intake is low. As noted above, almost all participants in the two cohorts of pregnant women took prenatal supplements (containing between 200 and 600 IU of vitamin D), vitamin D supplements (up to 10,000 IU), or both. Although participants from the BHIP study consumed more vitamin D overall due to higher supplement intake, they had similar 25-OHD concentrations to participants in the FAMILY study. Participants in the FAMILY study presented a broader range of 25-OHD concentrations, likely due to the larger sample size of FAMILY as compared with the BHIP study, with participants at the extremes with either clinical deficiency or excessive 25-OHD concentration. These results also suggest that sun exposure plays an important role through cutaneous production of vitamin D, ensuring most participants achieved an adequate vitamin D status. 

The average serum 25-OHD in our participants was moderately higher than that reported since 2000 for Canadian pregnant women in other provinces. In the Vancouver area, pregnant women predominately of European descent (*N* = 336 at 20–35 weeks gestation) had a mean (95% CI) 25-OHD of 66.7 (64.2–69.1) nmol/L [34]. In a larger study in Québec City and Halifax (*N* = 1635 at 12–15 weeks gestation, primarily of European descent), the mean (SD) 25-OHD was 52.7 (16.9) nmol/L [38]. Further, in Edmonton and Calgary, Alberta (*N* = 537, primarily of European ancestry), the mean (SD) serum 25-OHD was 93.3 (25.6) nmol/L in the first trimester and 95.3 (25) nmol/L in the second trimester of pregnancy [36]. The 25-OHD concentration of women in our study is comparable to the first two studies but lower than reported by the APrON study in Alberta [36]. These discrepancies in 25-OHD concentration may be due to participants’ exposure to sun, the nature of the study samples, where multiethnic participants have lower 25-OHD concentration [34], and because those with the highest socioeconomic status have the highest 25-OHD concentration [36]. In addition, the largest consumers of multivitamin supplements are found in Alberta, while the lowest consumers are in Quebec [30]. Discrepancies can also result from the methods used to measure serum 25-OHD—either by ELISA [34,38] or LC-MS/MS [36]. In all cohorts, the prevalence of deficiency was very low (either defined as 25-OHD < 25 nmol/L [38] or < 30 nmol/L [34,36]); from < 1 to 7% [34,36,38], aligning with our observed prevalence deficiency (defined as 25-OHD < 30 nmol/L) of 5% in FAMILY and <1% BHIP studies. The prevalence of insufficient 25-OHD (< 50 nmol/L) in these cohorts was between 2% and 45% [34,36,38], similar to what we observed in the FAMILY study (18%) and BHIP study (7%). 

Based on the contemporary studies noted above and using the IOM reference values, the majority of Canadian women have an adequate serum 25-OHD concentration in pregnancy, regardless of stage of pregnancy. However, controversy remains as to the ‘optimal’ 25-OHD concentration in pregnancy, since the clinical significance both for mother and infant of a 25-OHD above 50 nmol/L is still undefined [7,39,40,41]. Data from a recent systematic review suggests that pregnant women with bacterial vaginosis, gestational diabetes, pre-eclampsia and those with small for gestational age (SGA) babies have lower circulating 25-OHD than their healthy pregnant counterparts [42,43]. However, the value for what constitutes ‘lower 25-OHD’ was not defined in the review and their analysis included individual studies that used both 50 and 75 nmol/L as the cut-off for sufficiency. Conversely, a further systematic review of randomized controlled trials found no clear evidence for maternal benefits or reduced incidence of pre-term birth with supplementation of 25-OHD. In this review, only eight out of 43 trials were categorized as having an overall low risk of bias [39]. Many of the trials included in this review were small and of low quality; therefore, more research is needed before recommendations on optimal vitamin D status in pregnancy can be made. Further, a large prospective cohort study from New Zealand found that serum 25-OHD concentrations in pregnant women were not associated with pre-eclampsia, SGA babies or pre-term birth; however, of note, this population was largely 25-OHD replete [44]. 

Controversy also exists as to the optimal target for serum 25-OHD in pregnancy. A higher maternal 25-OHD concentration at delivery has been associated with infant cord blood 25-OHD status, and maternal use of vitamin D supplements was associated with higher odds of reaching sufficiency (defined as >75 nmol/L) for both mothers and infants [45]. However, uncertainty exists as to whether a higher maternal 25-OHD concentration (i.e., 25-OHD > 75 nmol/L) in pregnancy is linked with health benefits. It has been hypothesized that the optimal 25-OHD concentration would be the one leading to maximal conversion to the active form of vitamin D [46]. To that effect, data from one recent randomized trial were interpreted to indicate the total circulating 25-OHD must reach 100 nmol/L in order to optimize circulating 1,25-OH_2_D in pregnancy [47]. In that case, only 21% of participants in our cohort would have met or exceeded this 25-OHD concentration. In contrast to benefits, high maternal, cord and infant 25-OHD concentrations may have disadvantageous effects on infant growth. In an RCT including 798 mother and infant dyads, mothers with pregnancy 25-OHD > 125 nmol/L had the smallest infants at 6 months [48]. Further, an evidence-based consensus statement determined that there is little evidence for any benefits of maternal vitamin D supplementation on early life anthropometry and growth in the offspring or on clinical benefits for the mother [49]. It was concluded that the cut-off for vitamin D sufficient status of 50 nmol/L, as suggested by the DRI, remains the accepted standard [49]. Based on our data, with the current level of vitamin D fortification in Canada the use of supplements might be essential for pregnant women to reach the EAR for vitamin D, but is not the most important factor in ensuring participants have adequate vitamin D status. Summer season, and by inference, amount of sun exposure, appeared to have the strongest impact on maternal serum 25-OHD concentration in our cohorts. 

The factors that were significantly associated with maternal 25-OHD concentrations in this study are in agreement with reported maternal factors in pregnant women across Canada (Vancouver, Halifax and Québec City, Edmonton and Calgary). Summer season at time of blood draw [34,38] and being of European ancestry [34] were reported as significant factors associated with 25-OHD concentration in Canadian pregnant women living in Southern Ontario. While a pre-pregnancy BMI < 25 kg/m^2^ was associated with a higher maternal 25-OHD concentration compared to pre-pregnancy BMI ≥ 35 kg/m^2^ [38]; a relationship between pre-pregnancy BMI and maternal 25-OHD was observed in the FAMILY study but not in the BHIP study. This may relate to the larger sample size in FAMILY. Consumption of milk, a mandatory vitamin D-fortified food in Canada, was surprisingly not associated with maternal 25-OHD concentrations in either study. Health Canada recently indicated plans to increase the amount of vitamin D for mandatory fortification of milk and margarine in an effort to help Canadians meet the DRIs [50]. Until that comes into effect by the end of 2022, it is likely that milk consumption alone will not be sufficient source of vitamin D intake for Canadian pregnant women.

Our study has several strengths including a detailed dietary intake including food and supplement sources and measurement of serum 25-OHD concentrations by the gold standard LC-MS/MS [51]. The generalizability of the findings may be limited due to the demographic homogeneity (primarily of European descent, highly educated women). Another limitation includes the reporting bias inherent to diet records, but our dietary assessment was combined with direct measurement of nutritional status, providing a better evaluation of nutritional adequacy. Lastly, the lack of quantitative measurement of sun exposure limits the interpretation of the results, as there might be differences between an individual’s exposure due to variable time spent in outdoor activities, clothing coverage and use of sunscreen. Future steps include a prospective longitudinal assessment of participants in the BHIP study to investigate the association of maternal 25-OHD concentrations with pregnancy and neonatal health outcomes.

## Figures and Tables

**Table 1:** Demographic, lifestyle and physical characteristics of participants during pregnancy.

	FAMILY Study *N* = 332		BHIP Study *N* = 191	
Maternal Characteristics	*N*	(%)	*N*	(%)
Gestational age	24–36 weeks		12–17 weeks	
Pre-pregnancy BMI (kg/m^2^)				
Underweight (<18.5)	4	1	3	1
Normal weight (18.5–24.9)	144	45	91	48
Overweight (25.0–29.9)	103	32	61	32
Obese (≥30)	69	22	36	19
Unknown	12	-	0	-
Ethnicity				
European descent	285	86	171	90
Other	47	14	20	10
Household income (CAD)				
<$50,000	68	21	15	8
$50,000–$99,999	138	43	91	48
≥$100,000	111	35	79	41
Unknown	15	1	6	3
Education (years)				
≤13	46	14	0	0
>13	286	86	191	100
Smoking status				
Smoked during pregnancy	10	3	0	0
Former smoker; quit before pregnancy	107	33	n/a	n/a
Never smoked	212	64	n/a	n/a
Unknown	3	-	n/a	n/a
Exercise at study entry				
Not currently exercising	49	15	42	22
Currently exercising	283	85	147	77
Missing data	0	-	2	1

Data not applicable (n/a) as smoking status was an exclusion criteria in the BHIP study, and such data was not collected.

**Table 2:** Maternal serum 25-OHD concentration by season of blood draw and pre-pregnancy body mass index (BMI) category.

	FAMILY Study			BHIP Study		
**Category**	Serum 25-OHD, nmol/L Mean (SD) (95% CI)	*N* (%)	*p*-value	Serum 25-OHD, nmol/L Mean (SD) (95% CI)	*N* (%)	*p*-value
All participants	76.5 (32.9) (72.9, 80.1)	332	-	79.7 (22.3) (76.5, 82.9)	191	-
Season of blood draw			0.0001			0.0002
Summer (May–Oct.)	83.47 (34.3) (78.3, 88.7)	169 (51)		84.9 (21.0) (80.9, 89.0)	106 (55)	
Winter (Nov–Apr.)	68.5 (29.3) (63.9, 73.1)	160 (48)		73.2 (22.2) (68.4, 78.0)	85 (45)	
Missing data	-	3 (1)		-	-	
Pre-pregnancy BMI (kg/m^2^)			0.11			0.10
Underweight (<18.5)	72.2 (44.3) (1.6, 142.8)	4 (1)		90.3 (15.1) (52.6, 127.9)	3 (1)	
Normal (18.5–24.9)	79.5 (33.4) (74.0, 85.0)	144 (43)		82.2 (21.2) (77.8, 86.5)	93 (49)	
Overweight (25.0–29.9)	78.3 (33.7) (71.7, 84.9)	103 (31)		73.8 (20.5) (68.4, 79.2)	58 (31)	
Obese (≥30)	68.1 (30.0) (60.9, 75.3)	69 (21)		81.8 (26.6) (90.7, 73,0)	37 (19)	
Missing data	-	12 (4)		-	-	

**Table 3:** Multivariable analysis of factors associated with maternal serum 25-OHD concentrations during pregnancy in the FAMILY and BHIP studies.

Variables	FAMILY Study			BHIP Study		
	Estimated Coefficient	95% CI	*p*-Value	Estimated Coefficient	95% CI	*p*-Value
Ethnicity (European descent as reference)	**−5.85**	**−10.97, −0.72**	**0.025**	−5.91	−12.44, 0.61	0.075
Season of blood draw for baseline blood (Winter as reference)	**7.73**	**4.27, 11.18**	**<0.001**	**8.27**	**4.44, 12.09**	**<0.001**
Exercising at enrollment	0.53	−5.73, 4.66	0.840	3.08	−1.72, 7.88	0.206
Pre-pregnancy BMI	**−0.92**	**−1.52, −0.31**	**0.003**	−0.37	−1.19, 0.46	0.381
Total vitamin D intake	−0.01	−0.04, 0.02	0.469	−0.01	−0.04, 0.02	0.451
Vitamin D intake from supplement	0.01	−0.02, 0.05	0.422	0.02	−0.01, 0.05	0.174
Vitamin D intake from milk	1.03	−3.97, 6.02	0.687	0.03	−0.03, 0.10	0.272
Vitamin D intake from low fat dairy products	4.19	−0.43, 8.82	0.076	-	-	-
Vitamin D intake from regular fat dairy products (sour cream, cream, ice cream, cheese)	−2.11	−5.99, 1.77	0.285	**0.44**	**0.08, 0.81**	**0.017**

Bold format for significant results.

## References

[1] Heyden E.L., Wimalawansa S.J. (2017). Vitamin D: Effects on human reproduction, pregnancy, and fetal well-being. J. Steroid Biochem. Mol. Biol..

[2] Larqué E., Morales E., Leis R., Blanco-Carnero J.E. (2018). Maternal and foetal health implications of vitamin D status during pregnancy. Ann. Nutr. Metab..

[3] Curtis E.M., Moon R.J., Harvey N.C., Cooper C. (2018). Maternal vitamin D supplementation during pregnancy. Br. Med. Bull..

[4] Kovacs C.S. (2015). Calcium, phosphorus, and bone metabolism in the fetus and newborn. Early Hum. Dev..

[5] Thorne-Lyman A., Fawzi W.W. (2012). Vitamin D during pregnancy and maternal, neonatal and infant health outcomes: A systematic review and meta-analysis. Paediatr. Perinat. Epidemiol..

[6] Bi W.G., Nuyt A.M., Weiler H., Leduc L., Santamaria C., Wei S.Q. (2018). Association between vitamin D supplementation during pregnancy and offspring growth, morbidity, and mortality. JAMA Pediatr..

[7] De-Regil L., Palacios C., Lombardo L., Peña-Rosas J. (2016). Vitamin D supplementation for women during pregnancy. Cochrane Database Syst. Rev..

[8] Viljakainen H.T., Saarnio E., Hytinantti T., Miettinen M., Surcel H., Mäkitie O., Andersson S., Laitinen K., Lamberg-Allardt C. (2010). Maternal vitamin D status determines bone variables in the newborn. J. Clin. Endocrinol. Metab..

[9] Javaid M.K., Crozier S.R., Harvey N.C., Gale C.R., Dennison E.M., Boucher B.J., Arden N.K., Godfrey K.M., Cooper C., Princess Anne Hospital Study G. (2006). Maternal vitamin D status during pregnancy and childhood bone mass at age 9 years: A longitudinal study. Lancet.

[10] Viljakainen H.T., Korhonen T., Hytinantti T., Laitinen E.K.A., Andersson S., Mäkitie O., Lamberg-Allardt C. (2011). Maternal vitamin D status affects bone growth in early childhood—A prospective cohort study. Osteoporos. Int..

[11] Zhu K., Whitehouse A.J., Hart P.H., Kusel M., Mountain J., Lye S., Pennell C., Walsh J.P. (2014). Maternal vitamin D status during pregnancy and bone mass in offspring at 20 years of age: A prospective cohort study. J. Bone Min. Res..

[12] Godel J.C., Canadian Paediatric Society, First Nations, Inuit and Métis Health Committee (2007). Vitamin D supplementation: Recommendations for Canadian mothers and infants. Paediatr. Child Health.

[13] Hollis B.W. (2011). Vitamin D supplementation during pregnancy: Double-blind, randomized clinical trial of safety and effectiveness. J. Bone Miner. Res..

[14] Ross A., Taylor C., Yaktine A. (2011). Dietary Reference Intakes for Vitamin D and Calcium.

[15] Scientific Advisory Committee on Nutrition (2016). Vitamin D and Health.

[16] (2016). EFSA Panel on Dietetic Products Nutrition and Allergies (NDA) Dietary reference values for vitamin D. EFSA J..

[17] Morrison K.M., Atkinson S.A., Yusuf S., Bourgeois J., McDonald S., McQueen M.J., Persadie R., Hunter B., Pogue J., Teo K. (2009). The Family Atherosclerosis Monitoring In earLY life (FAMILY) study. Rationale, design, and baseline data of a study examining the early determinants of atherosclerosis. Am. Heart J..

[18] Perreault M., Atkinson S.A., Mottola M.F., Phillips S.M., Bracken K., Hutton E.K., Xie F., Meyre D., Morassut R.E., Prapavessis H. (2018). Structured diet and exercise guidance in pregnancy to improve health in women and their offspring: Study protocol for the Be Healthy in Pregnancy (BHIP) randomized controlled trial. Trials.

[19] Health Canada Prenatal Nutrition Guidelines for Health Professionals, Gestational Weight Gain. http://www.hc-sc.gc.ca/fn-an/alt_formats/pdf/nutrition/prenatal/ewba-mbsa-eng.pdf2010.

[20] Anand S.S., Yusuf S., Vuksan V., Devanesen S., Montague P., Kelemen L., Bosch J., Sigouin C., Teo K.K., Lonn E. (1998). The Study of Health Assessment and Risk in Ethnic groups (SHARE): Rationale and design. The SHARE Investigators. Can. J. Cardiol..

[21] Kelemen L.E., Anand S.S., Vuksan V., Yi Q., Teo K.K., Devanesen S., Yusuf S., Investigators S. (2003). Development and evaluation of cultural food frequency questionnaires for South Asians, Chinese, and Europeans in North America. J. Am. Diet. Assoc..

[22] Merchant A.T., Kelemen L.E., De Koning L., Lonn E., Vuksan V., Jacobs R., Davis B., Teo K.K., Yusuf S., Anand S.S. (2008). Interrelation of saturated fat, trans fat, alcohol intake, and subclinical atherosclerosis. Am. J. Clin. Nutr..

[23] Mottola M.F., Giroux I., Gratton R., Hammond J.A., Hanley A., Harris S., McManus R., Davenport M.H., Sopper M.M. (2010). Nutrition and exercise prevent excess weight gain in overweight pregnant women. Med. Sci. Sport. Exerc..

[24] Held C., Iqbal R., Lear S.A., Rosengren A., Islam S., Mathew J., Yusuf S. (2012). Physical activity levels, ownership of goods promoting sedentary behaviour and risk of myocardial infarction: Results of the INTERHEART study. Eur. Heart J..

[25] Davies G., Wolfe L., Mottola M., MacKinnon C. (2003). Joint SOGC/CSEP clinical practice guideline: Exercise in pregnancy and the postpartum period. Can. J. Appl. Physiol..

[26] The Analysis of 25-Hydroxyvitamin D in Serum Using UPLC/MS/MS. http://www.waters.com/webassets/cms/library/docs/720002748_vit_d_application_note.pdf.

[27] Hymøller L., Jensen S.K. (2011). Vitamin D analysis in plasma by high performance liquid chromatography (HPLC) with C30 reversed phase column and UV detection—Easy and acetonitrile-free. J. Chromatogr. A.

[28] Perichart-Perera O., Muñoz-Manrique C., Reyes-López A., Tolentino-Dolores M., Espino Y Sosa S., Ramírez-González M.C. (2017). Metabolic markers during pregnancy and their association with maternal and newborn weight status. PLoS ONE.

[29] Roth D.E., Abrams S.A., Aloia J., Bergeron G., Bourassa M.W., Brown K.H., Calvo M.S., Cashman K.D., Combs G., De-Regil L.M. (2018). Global prevalence and disease burden of vitamin D deficiency: A roadmap for action in low- and middle-income countries. Ann. N. Y. Acad. Sci..

[30] Langlois K., Greene-Finestone L., Little J., Hidiroglou N., Whiting S. (2010). Vitamin D status of Canadians as measured in the 2007 to 2009 Canadian Health Measures Survey. Stat. Can..

[31] Moon R.J., Harvey N.C., Cooper C., D ’angelo S., Crozier S.R., Inskip H.M., Schoenmakers I., Prentice A., Arden N.K., Bishop N.J. (2016). Determinants of the maternal 25-hydroxyvitamin D response to vitamin D supplementation during pregnancy. J. Clin. Endocrinol. Metab..

[32] Morisset A.-S., Weiler H.A., Dubois L., Ashley-Martin J., Shapiro G.D., Dodds L., Massarelli I., Vigneault M., Arbuckle T.E., Fraser W.D. (2016). Rankings of iron, vitamin D, and calcium intakes in relation to maternal characteristics of pregnant Canadian women. Appl. Physiol. Nutr. Metab..

[33] Dubois L., Diasparra M., Bédard B., Colapinto C.K., Fontaine-Bisson B., Tremblay R.E., Fraser W.D. (2018). Adequacy of nutritional intake during pregnancy in relation to prepregnancy BMI: Results from the 3D Cohort Study. Br. J. Nutr..

[34] Li W., Green T.J., Innis S.M., Barr S.I., Whiting S.J., Shand A., von Dadelszen P. (2011). Suboptimal vitamin D levels in pregnant women despite supplement use. Can. J. Public Health.

[35] Gomez M.F., Field C.J., Olstad D.L., Loehr S., Ramage S., Mccargar L.J., Kaplan B.J., Dewey D., Bell R.C., Bernier F.P. (2015). Use of micronutrient supplements among pregnant women in Alberta: Results from the Alberta Pregnancy Outcomes and Nutrition (APrON) cohort. Matern. Child Nutr..

[36] Aghajafari F., Field C.J., Kaplan B.J., Rabi D.M., Maggiore J.A., O’Beirne M., Hanley D.A., Eliasziw M., Dewey D., Weinberg A. (2016). The current recommended vitamin D intake guideline for diet and supplements during pregnancy is not adequate to achieve vitamin D sufficiency for most pregnant women. PLoS ONE.

[37] Savard C., Lemieux S., Weisnagel S.J., Fontaine-Bisson B., Gagnon C., Robitaille J., Morisset A.S. (2018). Trimester-specific dietary intakes in a sample of French-Canadian pregnant women in comparison with national nutritional guidelines. Nutrients.

[38] Woolcott C.G., Giguère Y., Weiler H.A., Spencer A., Forest J.C., Armson B.A., Dodds L. (2016). Determinants of vitamin D status in pregnant women and neonates. Can. J. Public Health.

[39] Roth D.E., Leung M., Mesfin E., Qamar H., Watterworth J., Papp E. (2017). Vitamin D supplementation during pregnancy: State of the evidence from a systematic review of randomised trials. BMJ.

[40] Eggemoen Å.R., Jenum A.K., Mdala I., Knutsen K.V., Lagerlov P., Sletner L. (2017). Vitamin D levels during pregnancy and associations with birth weight and body composition of the newborn: A longitudinal multiethnic population-based study. Br. J. Nutr..

[41] Harvey N.C., Holroyd C., Ntani G., Javaid K., Cooper P., Moon R., Cole Z., Tinati T., Godfrey K., Dennison E. (2014). Vitamin D supplementation in pregnancy: A systematic review. Health Technol. Assess..

[42] Aghajafari F., Nagulesapillai T., Ronksley P.E., Tough S.C., O’Beirne M., Rabi D.M. (2013). Association between maternal serum 25-hydroxyvitamin D level and pregnancy and neonatal outcomes: Systematic review and meta-analysis of observational studies. BMJ.

[43] Wei S., Qi H., Luo Z., Fraser W. (2013). Maternal vitamin D status and adverse pregnancy outcomes: A systematic review and meta-analysis. J. Matern. Neonatal Med..

[44] Boyle V.T., Thorstensen E.B., Mourath D., Jones M.B., McCowan L.M.E., Kenny L.C., Baker P.N. (2016). The relationship between 25-hydroxyvitamin D concentration in early pregnancy and pregnancy outcomes in a large, prospective cohort. Br. J. Nutr..

[45] Aghajafari F., Field C.J., Kaplan B.J., Maggiore J.A., O’Beirne M., Hanley D.A., Eliasziw M., Dewey D., Ross S., Rabi D. (2017). The high prevalence of vitamin D insufficiency in cord blood in Calgary, Alberta (APrON-D Study). J. Obstet. Gynaecol. Can..

[46] Wagner C.L., Hollis B.W. (2018). The Implications of Vitamin D Status During Pregnancy on Mother and her Developing Child. Front. Endocrinol..

[47] Wagner C.L., Taylor S.N., Dawodu A., Johnson D.D., Hollis B.W. (2012). Vitamin D and its role during pregnancy in attaining optimal health of mother and fetus. Nutrients.

[48] Hauta-alus H.H., Kajantie E., Holmlund-Suila E.M., Rosendahl J., Valkama S.M., Enlund-Cerullo M., Helve O.M., Hytinantti T.K., Viljakainen H., Andersson S. (2019). High pregnancy, cord blood and infant vitamin D concentrations may predict slower infant growth. J. Clin. Endocrinol. Metab..

[49] Munns C.F., Shaw N., Kiely M., Specker B.L., Thacher T.D., Ozono K., Michigami T., Tiosano D., Mughal M.Z., Makitie O. (2016). Global consensus recommendations on prevention and management of nutritional rickets. Horm. Res. Paediatr..

[50] Governement of Canada Summary of Proposed Amendments, Part I: Nutrition Symbols, Other Labelling Provisions, Partially Hydrogenated Oils and Vitamin D. https://www.canada.ca/en/health-canada/programs/consultation-front-of-package-nutrition-labelling-cgi/summary-of-proposed-amendments.html.

[51] Zerwekh J.E. (2008). Blood biomarkers of vitamin D status. Am. J. Clin. Nutr..

